# The Relationship Between Watching Baseball Games at a Home Stadium and Team Identification With Subjective Well‐Being Among Middle‐Aged and Older Baseball Fans

**DOI:** 10.1155/jare/8821334

**Published:** 2026-01-11

**Authors:** Jun Nakahara

**Affiliations:** ^1^ School of Contemporary Sociology, Chukyo University, Aichi, Toyota, Japan, chukyo-u.ac.jp

**Keywords:** identity theory, social identity theory, subjective well-being, team identification, watching baseball games

## Abstract

**Aim:**

This study examined the relationship between attending baseball games at a home stadium and team identification (including role team identification, group team identification, and fan community identification) with a professional Japanese baseball team, as well as subjective well‐being (comprising positive affect, negative affect, and life satisfaction) among middle‐aged and older fans of the Chunichi Dragons.

**Methods:**

A cross‐sectional online survey was conducted to collect data from 675 Japanese middle‐aged and older fans of the Chunichi Dragons (334 men and 341 women; mean age = 59.34 ± 10.79 years) residing in any of three Tokai prefectures (Aichi, Gifu, and Mie). The analysis items included the frequency of attending baseball games at the Vantelin Dome Nagoya (VDN), overall satisfaction with watching baseball games at VDN, team identification, and subjective well‐being.

**Results:**

Regression analysis revealed an inverted U‐shaped curvilinear relationship between life satisfaction and role team identification (squared term *B* = −0.092, *p* = 0.039), a positive relationship between fan community identification and life satisfaction (*B* = 0.278, *p* < 0.001), and a positive relationship between group team identification and negative affect (*B* = 0.240, *p* < 0.001). General satisfaction with watching baseball games at VDN was related to life satisfaction (*B* = 0.112, *p* = 0.043), positive affect (*B* = 0.138, *p* < 0.001), and negative affect (*B* = −0.079, *p* = 0.042); however, the frequency of attending baseball games at VDN was not associated with subjective well‐being.

**Conclusion:**

Spectator satisfaction was positively associated with subjective well‐being, whereas the relationship between team identification and subjective well‐being varies based on the types of team identification.

## 1. Introduction

Gerontological studies have demonstrated that leisure activities—defined as pleasurable activities that individuals engage in voluntarily when they are free from the demands of work or other responsibilities [1]—have beneficial effects on subjective well‐being among middle‐aged and older adults [1–4]. Particularly, subjective well‐being has been consistently linked to physically active leisure (i.e., playing sports) based on data from older adults in various countries [5–8], whereas there is less evidence for passive leisure activities (i.e., watching TV) [7]. A comparative study of the effects of active and passive leisure activities found that older adults who engaged in active leisure activities had higher subjective well‐being than their counterparts who engaged in passive leisure activities [9].

In recent years, the impact of watching sports as a leisure activity on subjective well‐being has been attracting attention. For example, sports spectating was positively related to life satisfaction in a study using U.S. and Australian samples [10], and watching local professional baseball games at a stadium also increased subjective happiness [11] and reduced depression symptoms in Japanese older adults [12]. Basically, the mechanism of the association between sports spectatorship and subjective well‐being was interpreted from the perspective of the social identity approach (SIA), emphasizing a sense of belonging to a group and interaction with other group members [10, 13]. These studies also focus on sports spectating in the field, which can be classified as an active leisure activity involving interaction with others and physical activity. Consequently, this study examined the relationship between watching professional baseball games at a stadium as an active leisure activity and subjective well‐being from the psychological perspective of SIA.

People often refer to a professional sports team to describe themselves, such as “I am a fan of Chunichi Dragons (a Japanese professional baseball team located in Aichi prefecture).” This statement is an example of team identity: a self‐concept based on the team‐related group membership, according to social identity theory [14]. Although team identification has been considered the spectators’ perceived connectedness to a team and the extent to which they experience the team’s successes and failures as their own, there have been confusions surrounding the concept and a lack of highly validated measures [15]. Recently, in this context, three components of identification (i.e., role team identification, group team identification, and fan community identification) were proposed, and their validated scales were developed after theoretically examining the details of identification in the field of sports management [15].

Role team identification is defined as the “importance of being a fan.” It is based on identity theory [16]. As individuals have many role‐based identities (e.g., mother, daughter, employee, and fan of a specific team), role team identification reflects the importance of one specific role identity as a fan of a sports team. The next two team identifications are based on social identity theory [14], related to group‐based identity (e.g., family, colleagues at work, and the fan community of a specific team). Group team identification is defined as “a state of mind in which fans incorporate their psychological affiliation with a particular sports team into their self‐concept and share in the team’s successes as well as their failures,” and fan community identification is “a sense of friendship that a spectator has with other spectators rooting for the same team.”

Several studies on sports management have shown that higher levels of team identification increase behavioral loyalty and commitment to the supported teams [17–19]; however, research addressing the relationship between team identification and well‐being is still in its infancy. One study did not indicate a direct effect of team identification on life satisfaction [10], and another study found a link between team identification and negative affect [20].

In the work context, identification with one’s own company promotes subjective well‐being and reduces psychological distress [21]. Further, this relationship is curvilinear (i.e., identification with the company increases workers’ subjective well‐being up to a certain degree but decreases at extremes) [21, 22]. That is, overly strong organizational identification can lead to overwork and work–family conflict among workers, ultimately reducing individual well‐being [23]. A previous study in the context of sports spectating demonstrated that soccer fans’ social identification could promote physical aggression [24]. Other research also suggests the hypothesis that low team status such as poor objective team performance reduces the positive linear relationship between team identification and the social aspect of life satisfaction [13]. Although this hypothesis was not empirically supported, it measured team performance over a relatively short period, such as a year, and did not address longer‐term downturns.

Fans who strongly identify with a team, especially during a long period of stagnation, might experience a decline in their well‐being along with repeated frustration. Consequently, this study aims to verify the association between watching baseball games at a home stadium and team identification with a Japanese professional baseball team on subjective well‐being (i.e., life satisfaction, positive affect, and negative affect) [25, 26] among middle‐aged and older Chunichi Dragons fans.

The Chunichi Dragons, a Japanese professional baseball team, have been in a slump in recent years, having last won the championship in 2011. In most seasons since then, they have finished either fifth or sixth out of six teams. Additionally, because the team has been continuously based in Nagoya, Aichi Prefecture, since its establishment in 1936, this study does not need to consider the complex effects of franchise relocation on the team identification of older fans [27]. Based on the above discussion, this study confirms the relationship between watching baseball games at a home stadium and subjective well‐being as well as tests the following hypothesis:

 Hypothesis 1: The relationship between each type of team identification (i.e., role team identification, group team identification, and fan community identification) and the subjective well‐being of Chunichi Dragons fans is curvilinear. That is, team identification levels, to a certain extent, would be positively associated with subjective well‐being; however, extremely high levels of team identification would be related to low subjective well‐being.

## 2. Methods

### 2.1. Participants

A cross‐sectional online survey was conducted in October and November 2022, and data were collected from 675 Japanese middle‐aged and older Chunichi Dragons fans, aged 40–70 years (mean age = 59.34 ± 10.79 years), who lived in one of three Tokai prefectures (Aichi, Gifu, and Mie), which are relatively close to the team’s home in Nagoya and are easily accessible for watching games at the home stadium (Vantelin Dome Nagoya [VDN]). Participants had visited VDN at least once in the past five years.

To minimize the impact of sex and age bias on the analysis, participants were stratified by both variables. Consequently, this study included 84 men and 87 women in their 40 s, 84 men and 82 women in their 50 s, 82 men and 85 women in their 60 s, and 84 men and 87 women in their 70 s. All participants eligible under the above criteria were recruited through INTAGE Inc., a Japanese research outsourcing company. Therefore, informed consent was obtained from the participants by INTAGE Inc. In addition, only those who have agreed to the survey contents and the research ethics guidelines listed on the top page of the online survey form responded to the online survey.

### 2.2. Survey Items

#### 2.2.1. Watching Baseball Games at a Home Stadium

This survey asked for the frequency of attending baseball games at VDN during the 2022 season and their general satisfaction at that time. Participants selected the number of times they watched baseball games at VDN in 2022 from six options (1 = none, 2 = 1 time, 3 = 2 times, 4 = 3 times, 5 = 4 times, 6 = 5 times or more) and responded on a five‐point Likert‐type scale regarding their general satisfaction with watching baseball games at VDN (1 = strongly disagree; 5 = strongly agree).

#### 2.2.2. Team Identification

All types of team identification were assessed using the scale developed in a previous study [15]. It comprised three items for role team identification (e.g., I consider myself to be a “real” fan of “team name”), five items for group team identification (e.g., “Team name’s” successes are my successes), and three items for fan community identification (e.g., I really sympathize with other “team name” fans who support “team name”). In this study, it was used after changing “team name” to Chunichi Dragons. Participants responded using a five‐point Likert scale (1 = strongly disagree; 5 = strongly agree). Each item score was summed and then divided by the number of items (range = 1–5). Cronbach’s αs for role team identification, group team identification, and fan community identification were 0.868, 0.855, and 0.874, respectively, indicating excellent internal consistency.

#### 2.2.3. Subjective Well‐Being

Life satisfaction, positive affect, and negative affect were measured as variables of subjective well‐being. Life satisfaction was measured using the Satisfaction with Life Scale [26, 28]. It comprises five items (e.g., In most ways, my life is close to my ideal), and participants responded on a seven‐point Likert‐type scale (1 = strongly disagree; 7 = strongly agree). The Japanese short version of the Affective Well‐Being Scale was used to assess participants’ positive and negative affect [29]. This measure was translated into Japanese from the affect scale that was originally developed by the Midlife in the United States survey [30]. A 30‐day response frame was used for both affect measures to capture more generalized affect, with positive affect assessed by three items (e.g., thoughtful) and negative affect assessed by four items (e.g., hopeless). The response options for the affect scale are as follows: 1 = none of the time, 2 = a little of the time, 3 = some of the time, 4 = most of the time, and 5 = all of the time. Each item score was summed and divided by the number of items (Range_life satisfaction_ = 1–7, Range_positive/negative affect_ = 1–5). Cronbach’s αs for life satisfaction, positive affect, and negative affect were 0.903, 0.858, and 0.811, respectively, indicating excellent internal consistency.

#### 2.2.4. Control Variables

This survey included sex, age, subjective health condition, subjective economic status, and living arrangement as control variables, as these indicators may be associated with both frequency of attending baseball games at VDN and subjective well‐being. Subjective health condition, subjective economic status, and living arrangement are associated with various types of leisure activities and life satisfaction [31–34]. Subjective health conditions and economic status were measured using a five‐point Likert scale (subjective health condition: 1 = very bad, 5 = very good; subjective economic status: 1 = having no financial leeway; 5 = having financial leeway). Regarding living arrangement, participants were categorized as living alone or not.

### 2.3. Analysis

Hierarchical regression models were estimated separately for each dependent variable to test the associations between watching baseball games at a home stadium, team identification, and the three types of subjective well‐being after all variables had been centered. Model 1 included only demographic controls (sex, age, subjective health status, subjective economic status, and living arrangements). Model 2 included the frequency of attending baseball games at VDN and general satisfaction with watching baseball games at VDN as independent variables. Model 3 included team identifications, and Model 4 examined their squared terms. The effects of team identification were tested both as linear relationships and as curvilinear relationships.

Two‐tailed significance was set at *p* < 0.05. All statistical procedures were performed using HAD version 17_202 [35]. Additionally, there were seven missing values for age (1.04%), which was the largest number across all variables. Given that the number of missing values in the data was considered very small, the listwise method was used in all analyses.

## 3. Results

Table 1 presents descriptive information regarding all variables. The average ± SD scores for the frequency of attending baseball games at VDN and for general satisfaction with watching baseball games at VDN were 1.88 ± 1.42 and 3.22 ± 0.79, respectively. All team identification scores were slightly lower than those in a previous study that assessed the same scales of team identities using younger Japanese participants [15].

**Table 1:** Descriptive statistics.

	Mean ± SD or *n* (%)
Sex (Female)	341 (50.44)
Age (years)	59.34 ± 10.79
Subjective health status	3.72 ± 0.79
Subjective economic condition	2.87 ± 0.83
Living arrangement (living alone)	66 (9.67)
Frequency of attending baseball game at VDN	1.88 ± 1.42
General satisfaction with watching baseball games at VDN	3.22 ± 0.79
Role team identification	3.33 ± 0.97
Group team identification	2.69 ± 0.83
Fan community identification	2.96 ± 0.92
Life satisfaction	4.10 ± 1.25
Positive affect	3.09 ± 0.92
Negative affect	2.00 ± 0.85

*Note:* The correlation coefficients among all variables are presented in the supporting information.

Abbreviation: VDN, Vantelin Dome Nagoya.

**Figure 1:**
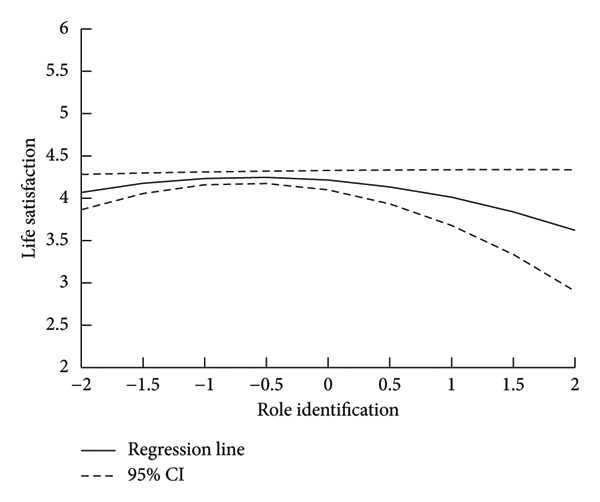
Curvilinear relationships between life satisfaction and role team identification. The solid line shows predicted trajectories of life satisfaction based on regression analysis. The dotted line indicates the 95% confidence interval.

Table 2 presents the results of the hierarchical regression models with life satisfaction as the dependent variable. Data in Table 2 show unstandardized regression coefficients, their 95% confidence intervals, standardized errors, and standardized coefficients in Model 4_LS_, as the R‐squared changes across all steps were significant (Δ*R* ^2^ range = 0.009–0.024, *p*s ranged = 0.001–0.020). The variance inflation factors (VIFs) in Model 4_LS_ ranged from 1.024 for living arrangement to 2.404 for group team identification. The association between general satisfaction with watching games at VDN and life satisfaction was significant (*B* = 0.112, *p* = 0.043). Regarding team identification, Figure 1 shows the regression curve of the relationship between role team identification and life satisfaction based on Model 4_LS_, as the squared term of role team identification (*B* = −0.092, *p* = 0.039) was significant in Model 4_LS_. The result showed a curvilinear relationship between role team identification and life satisfaction, as predicted, and the turning point was −0.614 for role team identification. In addition, a linear relationship between fan community identity and life satisfaction (*B* = 0.278, *p* < 0.001) was demonstrated.

**Table 2:** Results of the regression analysis on life satisfaction.

	Model 4_LS_			
	*B*	95% CI	SE	*β*
Intercept	4.214^∗∗^	4.099:4.329	0.06	
Watching baseball games at a home stadium				
Frequency of attending baseball game at VDN	−0.015	−0.077:0.048	0.032	−0.016
General satisfaction with watching baseball games at VDN	0.112^∗^	0.004:0.220	0.055	0.071
Team identification				
Role team identification	−0.113	−0.240:0.014	0.065	−0.087
Group team identification	−0.077	−0.228:0.075	0.077	−0.051
Fan community identification	0.278^∗∗^	0.142:0.414	0.069	0.206
Role team identification^2^	−0.092^∗^	−0.179:−0.005	0.045	−0.083
Group team identification^2^	0.061	−0.057:0.179	0.060	0.044
Fan community identification^2^	−0.082	−0.175:0.011	0.047	−0.075

*R* ^2^	0.283^∗∗^			

*Note:* Sex, age, subjective health condition, subjective economic status, and living arrangement were controlled. Model 4LS, Model 4 of regression analysis on life satisfaction. *B*, unstandardized coefficient; 95% CI, 95% confidence interval for *B*; *β*, standardized coefficient. All results, including regression coefficients for control variables, are presented in the supporting information.

Abbreviations: SE, standardized error; VDN, Vantelin Dome Nagoya.

^∗^ *p* < 0.05.

^∗∗^ *p* < 0.01.

The results of the regression models with positive affect as the dependent variable are presented in Table 3. Table 3 shows the results of Model 2_PA_, as the R‐square change from Models 2_PA_–3_PA_ was not significant (Δ*R* ^2^ = 0.008, *p* = 0.087), although the change from Models 1_PA_–2_PA_ was significant (Δ*R* ^2^ = 0.014, *p* = 0.004). The VIFs in Model 2_PA_ ranged from 1.021 for living arrangement to 1.116 for subjective economic status. Model 2_PA_ showed that general satisfaction with watching baseball games at VDN was related to positive affect (*B* = 0.138, *p* < 0.001), but the frequency of attending baseball games at VDN was not significant (*B* = 0.003, *p* = 0.901). These results indicated that only general satisfaction with baseball games at VDN was positively related to positive affect.

**Table 3:** Results of the regression analysis on positive affect.

	Model 2_PA_			
	*B*	95% CI	SE	*β*
Intercept	3.097^∗∗^	3.034:3.160	0.032	
Watching baseball games at a home stadium				
Frequency of attending baseball games at VDN	0.003	−0.043:0.048	0.023	0.004
General satisfaction with watching baseball games at VDN	0.138^∗∗^	0.056:0.220	0.042	0.118

*R* ^2^	0.205^∗∗^			

*Note:* Sex, age, subjective health condition, subjective economic status, and living arrangement were controlled. Model 2_PA_, Model 2 of regression analysis on positive affect. *B*, unstandardized coefficient; 95% CI, 95% confidence interval for *B*; *β*, standardized coefficient. All results, including regression coefficients for control variables, are presented in the supporting information.

Abbreviations: SE, standardized error; VDN, Vantelin Dome Nagoya.

^∗∗^ *p* < 0.01.

Finally, the results of the regression models with negative affect as the dependent variable are presented in Table 4. The change in R‐squared from Model 2_NA_ to Model 3_NA_ was significant, although the changes from Model 1_NA_ to Model 2_NA_ (*Δ* *R* ^2^ = 0.006, *p* = 0.087) and from Model 3_NA_ to Model 4_NA_ (Δ*R* ^2^ = 0.0003, *p* = 0.974) were not significant. The VIFs in Model 3_NA_ ranged from 1.023 for living arrangement to 2.401 for group team identification. In Model 3_NA_, general satisfaction with watching baseball games at VDN (*B* = −0.079, *p* = 0.042) and group team identification (*B* = 0.240, *p* < 0.001) were associated with negative affect, while role team identification (*B* = −0.070, *p* = 0.124) and fan community identification (*B* = −0.092, *p* = 0.056) were not related. In addition, the frequency of attending baseball games at VDN (*B* = 0.001, *p* = 0.982) was not linked to negative affect. These results clearly confirmed that individuals with higher levels of group team identification were more likely to experience negative affect.

**Table 4:** Results of the regression analysis on negative affect.

	Model 3_NA_			
	*B*	95% CI	SE	*β*
Intercept	1.993^∗∗^	1.935:2.051	0.029	
Watching baseball games at a home stadium				
Frequency of attending baseball games at VDN	0.001	−0.044:0.045	0.02	0.001
General satisfaction with watching baseball games at VDN	−0.079^∗^	−0.155:−0.003	0.04	−0.074
Team identification				
Role team identification	−0.070	−0.159:0.019	0.045	−0.080
Group team identification	0.240^∗∗^	0.133:0.347	0.055	0.237
Fan community identification	−0.092	−0.187:0.003	0.048	−0.101

*R* ^2^	0.211^∗∗^			

*Note:* Sex, age, subjective health condition, subjective economic status, and living arrangement were controlled. Model 3NA, Model 3 of regression analysis on negative affect. *B*, unstandardized coefficient; 95% CI, 95% confidence interval for *B*; *β*, standardized coefficient. All results, including regression coefficients for control variables, are presented in the supporting information.

Abbreviations: SE, standardized error; VDN, Vantelin Dome Nagoya.

^∗^ *p* < 0.05.

^∗∗^ *p* < 0.01.

## 4. Discussion

This study investigated the association between watching baseball games in a home stadium, team identification, and subjective well‐being among middle‐aged and older fans. From a series of analyses, the hypothesis was supported only in the relationship between role team identification and life satisfaction. That is, the relationship between role team identification and life satisfaction among Chunichi Dragons fans is curvilinear: At a certain level, role team identification was positively associated with life satisfaction, but conversely, extremely high levels of role team identification were related to low subjective well‐being. Middle‐aged and older fans with greater fan community identification reported greater life satisfaction, while those with greater team identification experienced more negative affect. Further, middle‐aged and older adults who are satisfied with watching professional baseball games at VDN have better subjective well‐being in all aspects.

As shown in Figure 1, life satisfaction was lower when role identification exceeded a certain level. According to identity theory, [16, 36] a highly important role identity for an individual is selected in various situations, and as a result, the individual is strongly motivated to act in ways that are consistent with the social behaviors expected of their selected role. It has also been explained in activity theory [37], a theory of gerontology, that having a role identity for older adults helps maintain a sense of self that is connected to society through social roles and contributes to their subjective well‐being. However, for fans with strong role team identification, the long‐standing poor performance of the Chunichi Dragons is likely to foster pessimistic behavior associated with that fandom (e.g., making offensive online posts, repeating pessimistic comments about the team’s future) and reduce life satisfaction. Table 2 shows the positive relationship between fan community identification and life satisfaction. Based on SIA [38, 39], individuals who identified more strongly with the fan community of the Chunichi Dragons could more easily access psychological resources, such as social support. As such resources enhance subjective well‐being, identification with the fan community would be a life‐enhancing experience with little or nothing to do with the team’s performance.

As shown in Tables 3 and 4, the results also indicated that fans with higher group team identification experienced negative affect more frequently, while this was not related to positive affect. Additionally, the other two types of team identification were not associated with affective well‐being. Affective well‐being is short‐term well‐being compared to life satisfaction, and the scale used in this study also assessed emotional states over the last 30 days [29]. Since the survey period for this study was at the end of the 2022 season and the number of games was small, there were few opportunities for fans to cheer in their role behavior and to interact with other fans during the games. That is why role team identification and fan community identification are not associated with affective well‐being. Because high group team identification suggests a strong tendency to incorporate the team’s successes and failures into one’s own [13, 15], fans with high group team identification might be more likely to develop negative feelings by reflecting on a one‐season slump of the Chunichi Dragons.

Although the frequency of attending baseball games at VDN was not associated with subjective well‐being, general satisfaction with watching baseball games at VDN was suggested to have a favorable impact on all aspects of subjective well‐being. As a previous study indicated, the direct impact of sports watching on subjective well‐being is extremely small when targeting fans of teams competing in the games [13, 20]. The frequency of attending baseball games at VDN and indicators of subjective well‐being were not considered related in this study either. Fans who have a favorite team may improve their subjective well‐being not by watching the games but by being satisfied with various aspects of the stadium experience, such as service, atmosphere, and team performance.

This study makes two academic contributions and provides implications for practice. First, to my knowledge, this is the first study to address the relationship between watching baseball games at a stadium as a leisure activity and the subjective well‐being of middle‐aged and older professional baseball fans in Japan. No direct effects of watching professional baseball games on subjective well‐being were found. However, the effects of general satisfaction with watching baseball games and team identification were confirmed. Second, this study analyzed the association between team identification and subjective well‐being using conceptually organized and reconstructed concepts and scales, whereas theoretical examinations of the constructs and the validity of the scales have been inadequate in the field of sports management research. These results suggest that the three types of team identification differ in their influence on subjective well‐being, and each psychological mechanism associated with the three types of team identification should be interpreted from each theoretical perspective. As a practical implication, since fan community identification showed a linear relationship with life satisfaction, events that strengthen the sense of community among fans are suggested to be effective in enhancing fans’ subjective well‐being. For example, wearing team‐specific items such as caps, wristbands, and uniforms featuring the same design can enhance fan community identification [18]. While enhancing the other two types of identification may contribute to improving behavioral loyalty [15], based on the results of this study, it does not lead to an improvement in subjective well‐being. Fan community identification may play a key role in both team management and in promoting the well‐being of middle‐aged and older fans.

The present study had some limitations. First, only Chunichi Dragons fans were surveyed. Owing to the team’s poor performance in recent years and its long‐standing roots in the community, this study was conducted with Chunichi Dragons fans. However, whether the impact of team identification on fans’ subjective well‐being is due to the weakness of the team they support cannot be determined without comparing data from fans who support teams that have performed well in recent years or by comparing Chunichi Dragons fan data during periods of good versus poor team performance within a single season. Therefore, it is necessary to examine the relationship between team identification and subjective well‐being through a survey of a larger number of team fans, as well as surveys conducted at various points throughout a single season. Second, several problems have been identified with online surveys, such as satisficing among online survey respondents [40]. As most sports management studies are conducted using similar methods [13, 20], this study’s results have a certain validity, but further field research will be necessary. Third, this study did not consider the impact of leisure activities or jobs other than watching professional baseball games. It is difficult to include all factors that are thought to influence subjective well‐being in a single study; however, this should be kept in mind when interpreting the results. Despite these limitations, this study provides new insights into the relationship between the subjective well‐being of middle‐aged and older fans and professional baseball games.

## Ethics Statement

This study was approved by the Ethics Committee of the School of Contemporary Sociology, Chukyo University (No. 2022‐092).

## Conflicts of Interest

The author declares no conflicts of interest.

## Author Contributions

The sole author has done all the activities and conceptions of the idea to the preparations for the final manuscript.

## Funding

The author received no specific funding for this work.

## Supporting Information

Correlation coefficients between all study variables and all results of multiple regression analyses with life satisfaction, positive affect, and negative affect as dependent variables.

## Supporting information


**Supporting Information** Additional supporting information can be found online in the Supporting Information section.

## Data Availability

The data that support the findings of this study are available from the corresponding author upon reasonable request.
