# Supplementary material for: The Relationship Between Watching Baseball Games at a Home Stadium and Team Identification With Subjective Well‐Being Among Middle‐Aged and Older Baseball Fans
Source: J Aging Res. 2026 Jan 11;2026:8821334. doi: 10.1155/jare/8821334 (PMC12791156; doi:10.1155/jare/8821334)
Supplement: Supplementary file 1 — Supporting Information Additional supporting information can be found online in the Supporting Information section. [file JARE-2026-8821334-s001.zip › Supporting information 1.pdf]

Supporting information 1

Appendix 1 Correlations among all study variables

	1	2	3	4	5	6	7	8	9	10	11	12
1. Sex (1 = Female)	—											
2. Age (years)	.001	—										
3. Subjective health status	.100 **	-.013	—									
4. Subjective economic condision	.111 **	.177 **	.237 **	—								
5. Living arrangement (1 = Living alone)	-.073	.058	-.066	-.083 *	—							
6. Frequency of attending baseball game at VDN	-.009	-.029	.063	.076 *	.009	—						
7. General satisfaction with watching baseball games at VDN	.119 **	.008	.135 **	.078 *	-.067	.129 **	—					
8. Role team identification	-.195 **	.097 *	.041	.038	.012	.322 **	.109 **	—				
9. Group team identification	-.065	.098 *	-.028	-.006	-.008	.144 **	.102 **	.652 **	—			
10. Fan community identification	-.007	.077 *	.046	.040	-.040	.188 **	.212 **	.617 **	.705 **	—		
11. Life satisfaction	.155 **	.177 **	.271 **	.421 **	-.134 **	.032	.163 **	.048	.062	.179 **	—	
12. Positive affect	.172 **	.165 **	.279 **	.349 **	-.091 *	.055	.187 **	.030	.029	.124 **	.690 **	—
13. Negative affect	.004	-.230 **	-.273 **	-.280 **	.038	-.034	-.124 **	-.043	.090 *	-.036	-.309 **	-.314 **

***p* < .01, **p* < .05

Appendix 2 All results of hierarchical regression analysis on life satisfaction

	Model 1_{LS}				Model 2_{LS}				Model 3_{LS}				Model 4_{LS}			
	B	*95% CI*	*SE*	*β*	*B*	*95% CI*	*SE*	*β*	*B*	*95% CI*	*SE*	*β*	*B*	*95% CI*	*SE*	*β*
Intercept	4.099 **	4.016 : 4.183	0.043		4.100 **	4.016 : 4.183	0.042		4.101 **	4.019 : 4.183	0.042		4.214 **	4.099 : 4.329	0.059	
Control variables																
Sex (1 = female)	0.227 **	0.058 : 0.396	0.086	0.091	0.203 *	0.034 : 0.372	0.086	0.081	0.172 *	0.000 : 0.344	0.088	0.069	0.148	-0.024 : 0.321	0.088	0.059
Age (years)	0.014 **	0.006 : 0.022	0.004	0.122	0.014 **	0.006 : 0.022	0.004	0.121	0.013 **	0.005 : 0.021	0.004	0.115	0.012 **	0.004 : 0.020	0.004	0.104
Subjective health condition	0.286 **	0.176 : 0.397	0.056	0.179	0.270 **	0.159 : 0.380	0.056	0.169	0.265 **	0.155 : 0.375	0.056	0.166	0.254 **	0.145 : 0.364	0.056	0.159
Subjective economic status	0.511 **	0.405 : 0.618	0.054	0.338	0.508 **	0.401 : 0.614	0.054	0.335	0.506 **	0.401 : 0.611	0.054	0.334	0.510 **	0.406 : 0.615	0.053	0.337
Living arrangement (1 = living alone)	-0.412 **	-0.697 : -0.126	0.145	-0.097	-0.392 **	-0.676 : -0.107	0.145	-0.093	-0.360 **	-0.641 : -0.079	0.143	-0.085	-0.349 *	-0.629 : -0.070	0.142	-0.083
Watching baseball games at a home stadium																
Frequency of attending baseball games at VDN					-0.017	-0.077 : 0.043	0.031	-0.019	-0.022	-0.085 : 0.040	0.032	-0.025	-0.015	-0.077 : 0.048	0.032	-0.016
General satisfation with watching baseball games at VDN					0.153 **	0.046 : 0.261	0.055	0.097	0.105	-0.004 : 0.213	0.055	0.066	0.112 *	0.004 : 0.220	0.055	0.071
Team identification																
Role team identification									-0.100	-0.226 : 0.027	0.064	-0.077	-0.113	-0.240 : 0.014	0.065	-0.087
Group team identification									-0.071	-0.223 : 0.081	0.077	-0.047	-0.077	-0.228 : 0.075	0.077	-0.051
Fan community identification									0.297 **	0.163 : 0.432	0.069	0.220	0.278 **	0.142 : 0.414	0.069	0.206
Role team identification^2													-0.092 *	-0.179 : -0.005	0.045	-0.083
Group team identification^2													0.061	-0.057 : 0.179	0.060	0.044
Fan community identification^2													-0.082	-0.175 : 0.011	0.047	-0.075
R^2		0.238 **				0.247 **				0.271 **				0.283 **		
R^2 *Change*						0.009 *				0.024 **				0.012 *		

B, unstandardized coefficient; *95% CI*, 95% confidence interval for B; *SE*, standardized error; *β*, standardized coefficient; VDN, Vantelin Dome Nagoya.

Model 1_{LS}-4_{LS}, Model 1-4 of regression analysis on life satisfaction

***p* < .01, **p* < .05
