# Supplementary material for: The Relationship Between Watching Baseball Games at a Home Stadium and Team Identification With Subjective Well‐Being Among Middle‐Aged and Older Baseball Fans
Source: J Aging Res. 2026 Jan 11;2026:8821334. doi: 10.1155/jare/8821334 (PMC12791156; doi:10.1155/jare/8821334)
Supplement: Supplementary file 1 — Supporting Information Additional supporting information can be found online in the Supporting Information section. [file JARE-2026-8821334-s001.zip › Supporting information 2.pdf]

Supporting information 2

Appendix 3 All results of hierarchical regression analysis on positive affect

	Model 1_{PA}				Model 2_{PA}			
	B	*95% CI*	*SE*	*β*	*B*	*95% CI*	*SE*	*β*
Intercept	3.097 **	3.033 : 3.160	0.032		3.097 **	3.034 : 3.160	0.032	
Controlled variables								
Sex (1 = Female)	0.209 **	0.079 : 0.337	0.066	0.113	0.188 **	0.059 : 0.316	0.065	0.101
Age (years)	0.011 **	0.005 : 0.017	0.003	0.126	0.011 **	0.005 : 0.017	0.003	0.126
Subjective health condition	0.236 **	0.152 : 0.320	0.043	0.200	0.220 **	0.135 : 0.304	0.043	0.186
Subjective economic status	0.295 **	0.214 : 0.377	0.041	0.264	0.289 **	0.208 : 0.370	0.041	0.258
Living arrangement (1 = Living alone)	-0.178	-0.396 : 0.040	0.111	-0.057	-0.161	-0.376 : 0.056	0.110	-0.051
Watching baseball games at a home stadium								
Frequency of attending baseball games at VDN					0.003	-0.043 : 0.048	0.023	0.004
General satisfation with watching baseball games at VDN					0.138 **	0.056 : 0.220	0.042	0.118
R^2		0.191 **				0.205 **		
R^2 *Change*						0.014 **		

B, unstandardized coefficient; *95% CI*, 95% confidence interval for B; *SE*, standardized error; *β*, standardized coefficient; VDN, Vantelin Dome Nagoya.
Model 1_{PA}-2_{PA}, Model 1-2 of regression analysis on positive affect
$^{**}p < .01$

Appendix 4 All results of hierarchical regression analysis on negative affect

	Model 1_{NA}				Model 2_{NA}				Model 3_{NA}			
	B	*95% CI*	*SE*	*β*	*B*	*95% CI*	*SE*	*β*	*B*	*95% CI*	*SE*	*β*
Intercept	1.992 **	1.933 : 2.051	0.030		1.992 **	1.933 : 2.050	0.042		1.993 **	1.935 : 2.051	0.029	
Controlled variables												
Sex (1 = Female)	0.091	-0.027 : 0.210	0.060	0.091	0.104	-0.015 : 0.222	0.086	0.061	0.097	-0.024 : 0.218	0.062	0.058
Age (years)	-0.016 **	-0.022 : -0.010	0.003	0.122	-0.016 **	-0.022 : -0.010	0.004	-0.204	-0.017 **	-0.022 : -0.011	0.003	-0.212
Subjective health condition	-0.269 **	-0.346 : -0.191	0.039	0.179	-0.259 **	-0.336 : -0.181	0.056	-0.240	-0.245 **	-0.322 : -0.168	0.039	-0.227
Subjective economic status	-0.208 **	-0.283 : -0.134	0.038	0.338	-0.204 **	-0.279 : -0.129	0.054	-0.200	-0.198 **	-0.272 : -0.124	0.038	-0.193
Living arrangement (1 = Living alone)	0.042	-0.159 : 0.242	0.102	-0.097	0.031	-0.169 : 0.231	0.145	0.011	0.032	-0.166 : 0.229	0.101	0.011
Watching baseball games at a home stadium												
Frequency of attending baseball games at VDN					-0.006	-0.048 : 0.036	0.021	-0.010	0.001	-0.044 : 0.045	0.022	0.001
General satisfation with watching baseball games at VDN					-0.083 *	-0.159 : -0.007	0.039	-0.078	-0.079 *	-0.155 : -0.003	0.039	-0.074
Team identification												
Role team identification									-0.070	-0.159 : 0.019	0.045	-0.080
Group team identification									0.240 **	0.133 : 0.347	0.055	0.237
Fan community identification									-0.092	-0.187 : 0.003	0.048	-0.101
R^2		0.180 **				0.187 **				0.211 **		
R^2 *Change*						0.007				0.024 **		

B, unstandardized coefficient; *95% CI*, 95% confidence interval for B; *SE*, standardized error; *β*, standardized coefficient; VDN, Vantelin Dome Nagoya.
Model 1_{NA}-3NA, Model 1-3 of regression analysis on negative affect
$^{**}p < .01$, $^{*}p < .05$
